# Microvascular engineering in perfusion culture: immunohistochemistry and CLSM findings

**DOI:** 10.1186/1746-160X-2-26

**Published:** 2006-08-16

**Authors:** Bernhard Frerich, Kerstin Zückmantel, Alexander Hemprich

**Affiliations:** 1 Department of Oral and Maxillofacial Surgery, Plastic Facial Surgery, University of Leipzig, Nürnberger Str. 57, D-04103 Leipzig, Germany

## Abstract

**Background:**

One of the most challenging problems in tissue engineering is the establishment of vascular supply. A possible approach might be the engineering of microvasculature in vitro and the supply by engineered feeder vessels.

**Methods:**

An in vitro model for a small-diameter vessel was developed and made from adipose tissue stromal cells and human umbilical vein endothelial cells in a tube-like gelatine scaffold. The number of "branches" emerging from the central lumen and the morphology of the central lumen of the vessel equivalent were assessed after 16 days of either pulsatile perfusion culture or culture in rotating containers by evaluation of immunohistochemically stained sections (n = 6 pairs of cultures). Intramural capillary network formation was demonstrated in five experiments with confocal laser scanning microscopy.

**Results:**

Perfused specimens showed a round or oval lumen lined by a single layer of endothelial cells, whereas following rotation culture the lumen tended to collapse. Confocal laser scanning microscopy showed more extended network formation in perfused specimens as compared to specimens after rotation culture. Partially highly interconected capillary-like networks were imaged which showed orientation around the central lumen. Perfused specimens exhibited significantly more branches emerging from the central lumen. There were, however, hardly any capillary branches crossing the whole vessel wall.

**Conclusion:**

Pulsatile perfusion supports the development of vascular networks with physiological appearance. Advances in reactor development, acquisition of functional data and imaging procedures are however necessary in order to attain the ultimate goal of a fully functional engineered supplying vessel.

## Background

Recent literature has focused increasingly on the issue of nutrition and oxygenation of larger tissue equivalents in tissue engineering [1-9]. Supply by diffusion does not exceed 100 to 300 μm in vivo as well as in vitro [10]. Consequently, there is an obvious need for a vascular network or an alternative equivalent.

The co-cultivation of endothelial cells and the cells of a target tissue has been proposed in order to accomplish immediately functioning vascularization [2,11]. Nevertheless, an appropriate solution for the interface between the artificial capillary network and the existing blood circulation of the recipient site has yet to be found. Many approaches proceed from the principle of free transplantation. Engineered microvessels of the tissue construct join with the surrounding microvasculature of the recipient site ("inosculation"). Another approach follows the principles of microsurgical transplantation. This comprises the inclusion of small diameter vessel substitutes ("donor vessels") into the artificial tissue, which might be able to supply the surrounding tissue. The small diameter vessel has to be suitable for the development of branches that enable a connection to the surrounding microvessels. While the engineering of a fully functional "feeder donor vessel" is the ultimate aim, the present study deals with the evolution of branches in a tube-like construct, which was designed as a preliminary in vitro model. The major concern was to demonstrate the engineering of capillary-like networks in vitro, which show patent branches to the central luminal compartment of the vessel model. An important aspect was the application on hydrodynamic forces, since it is known that endothelial cells as the interface between flowing blood and vessel wall are susceptible to different flow parameters [12-14].

## Methods

### Cells

Adipose tissue stromal cells (ATSC): Small pieces of adipose tissue (< 0.5 cm^3^) were collected from routine operations in the Department of Oral and Maxillofacial Surgery at the University of Leipzig. Informed consent was obtained from and signed by all patients. The processing and cultivation has been described earlier in detail [2,3]. The adipose tissue was minced with sterile scissors and subjected to collagenase digestion (collagenase type II, Boehringer, Mannheim, Germany). The suspension was filtrated over a 100 μm nylon mesh, centrifuged and plated on tissue culture flasks (Greiner, Frickenhausen, Germany). Cells were cultured at 37°C in a 5% humidified CO_2 _atmosphere. The culture medium (IMDM/Ham F-12 1:1 with 10% newborn calf serum (NCS), all from Life Technologie, Paisley, Scotland) was changed weekly. The cells were passaged in a 1:4 ratio. 3^rd ^and 4^th ^passage cells were used in the experiments. Flow cytometry showed that less than 0.5% (0.33 ± 0.23 %, mean ± standard deviation) of ATSC of six donors prepared in this way expressed CD31. Alpha-actin was expressed by 13.3 ± 8%, CD90 by 46.1 ± 25.7%, CD105 (SH2) by 23.8 ± 25.7%, CD73 (SH3) by 53 ± 39.2%, the latter are known to be positive in mesenchymal multi-lineage cells. The progenitor cell character also was proved by the ability to undergo adipogenic, osteogenic and smooth muscle differentiation (unpublished data).

Human umbilical vein endothelial cells (HUVEC): Umbilical cords were obtained from the Department of Gynaecology and Obstetrics of the University of Leipzig, clamped immediately and stored at 4°C in buffered saline until further processing. The umbilical vein was rinsed and filled with collagenase 0.1% (collagenase type II, Boehringer, Mannheim, Germany). A serum-supplemented medium was added and the resulting cell suspension centrifuged (300 g, 10 min.). The pellet was seeded on tissue culture flasks and cultivated in the incubator. Passages 3 to 4 were used for the experiments. The purity of the HUVEC was checked out by means of phase contrast morphology, DiI-Ac-LDL uptake and von Willebrand antigen staining.

### Fabrication of tube-like constructs

The fabrication of tube-like constructs has been described in detail elsewhere (Frerich et al submitted). A tube of 50 mm length was carved from commercially available, stiff gelatine sponge material (Spongostan, Johnson & Johnson, Norderstedt, Germany) with an inner lumen of 1 to 2 mm. After gas-sterilization, they were placed in rotating culture containers (In Vitro Systems and Services, Göttingen, Germany) and seeded with ATSC (one densely grown 75 cm^2 ^tissue culture flask, ca 10^7 ^cells) and HUVEC (ca. 10^6 ^cells). The seeding procedure was repeated three times in weekly intervals. Cells of different donors were allowed. Culture modules were placed on a roller unit and set on rotation with 10 rpm. The culture medium was IMDM/Ham F-12 1:1 with 10% NCS, supplemented with insulin and transferrin (all from Life Technologies, Paisley, Scotland). Medium was changed twice weekly. Two days prior to the start of the experiments, the inner lumen was lined additionally with endothelial cells as follows: HUVEC (ca. 10^6 ^cells) were trypsinized from a 75 cm^2 ^culture flask, centrifuged and re-suspended in a fibrin solution. The suspension was instilled into the tubes and a silicon mandrin (diameter 1 mm) placed into the lumen. The tubes were placed back in the culture module. By this procedure, also the outer surface was seeded with endothelial cells again. After 6 hours, the mandrin was removed. The following day, the tubes were ready for use in experiments.

### Perfusion vs. rotation experiments

**Figure 1:**
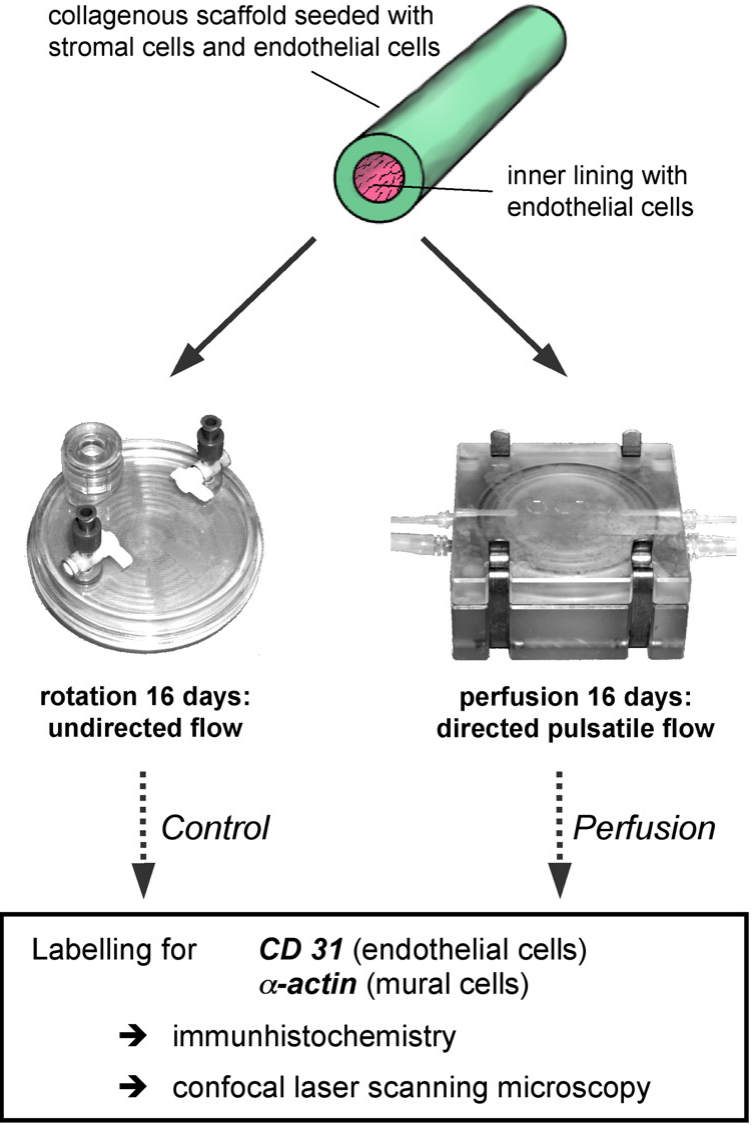
Experimental setting

The prepared tubes were divided into two pieces, one measuring a third and the other two-thirds of the length. The smaller part (a third of the length, ca. 1 – 1.5 cm) was placed back in the rotation culture container ("control group"). The longer part of the tube ("perfusion group", two-thirds of the length, ca. 2.5 cm) was placed in the minutissue perfusion chamber (gradient container, minucells and minutissue, Bad Abbach, Germany). This consisted of a 47 mm diameter chamber with two pairs of outlets located opposite each other. The lower pair was used to lead small silicon tubes into the chamber and the tubular construct was fixed with surgical sutures between these silicon tubes. Through this tubing, the vessel equivalent was perfused with medium using a roller pump. The medium perfused through the lumen of the vessel equivalent was collected and reused after filtration, whereas the medium "extravasating" to the extraluminal compartment was discarded (a quarter to a third of the perfused quantities). Further details of the system have been described elsewhere (Frerich et al. submitted). The experiments were conducted over a period of 16 days (see scheme in Fig. 1). During this time, the perfusion rate was raised from 100 μl/min to 500 μl/min and the pulse rate altered from 6/min to 16/min in 8 steps (every two days). The perfusion culture and the controls (rotation) were performed with a serum-free (five experiments) or serum-reduced (3%, one experiment) culture medium. In rotated controls, medium was changed twice weekly. Finally, the specimens from perfusion chamber and control group were harvested, fixed in formaldehyde 2%/paraformaldehyde 2% in PBS for 24 hours and cut in 2 mm cross- sectional slices. Two of these slices (from the middle portion of the specimen) were embedded in paraffin, a further was stained en bloc for laser scanning microscopy.

### Immunohistochemical staining and count of capillary branches

Histological sections were double-labelled with anti-CD31 (demonstration of endothelial cells) and anti-α-actin (demonstration of mural cells, i.e. pericytes and smooth muscle cells). After de-paraffination and rehydration, endogenous peroxidase activity was blocked (0.03% H_2_O_2_). The specimens were incubated with a mouse-anti-human CD31 antibody diluted 1:200 in PBS with 0.5% BSA and subsequently with an alkaline phosphatase-labelled goat-anti-mouse-polymer conjugate (EnVision AP). After having been covered with diluted mouse serum, the specimens were incubated with mouse-anti-human α-actin-EPOS/HRP conjugate (HRP-labelled polymer conjugated with anti-α-actin). Visualization was performed with BCIP/NBT substrate for the AP labelled structures (CD31 positivity) and DAB solution for the HRP conjugated α-actin-positive structures (all reagents from DAKO, Hamburg, Germany). Finally, the sections were counterstained with methyl green or nuclear fast red and embedded in DePeX (Serva, Heidelberg, Germany).

The lumen structure and the endothelial lining of the central lumen of the vessel equivalent were judged qualitatively. Entrances branching off from the central lumen to the capillary-like network in the vessel equivalent's wall were counted on each 8 histological sections and averaged. A SPSS statistical software package was utilised for statistical analysis. The difference in the number of entrances from the central lumen between the perfusion group and the control group was verified with the aid of the Mann Whitney U-test. The level of significance was set at p < 0.05.

### Confocal laser scanning microscopy

The visualisation of capillary-like structures with confocal laser scanning microscopy (CLSM) was performed in order to demonstrate three-dimensional network formation and presence of mural cells (pericytes and smooth muscle cells). UEA-I-lectin was used for demonstration of the capillary strains. Labelling with CD31 had been performed in parallel and showed the same results as labelling with UEA-I-lectin. Consequently, UEA-I-lectin could be considered as an endothelial cell specific marker in this co-culture model. Pericytes and smooth muscle cells were demonstrated by their content of α-actin in analogy to the immunohistochemical staining described above.

Specimens were fixed with formaldehyde/paraformaldehyde, cut into 1 mm slices and stained en bloc first with rhodamin-labelled UEA-I-lectin (Sigma-Aldrich GmbH, Steinheim, Germany). After rinsing they were additionally incubated with anti-α-actin (monoclonal mouse antibody, DAKO, Hamburg, Germany) and subsequently labelled with FITC-coupled goat anti mouse Fab'2 fragment. The tissue blocks were embedded in gelatine. The label was evaluated with a confocal laser-scanning microscope (Leica TCS 4D, Leica, Germany). In every specimen 6 to 12 fields were evaluated each of a size of 800 microns square and a maximum depth of 40 to 60 microns. Within this depth, fluorescence label proved to be constant and reliable. The images from green and from blue fluorescent excitation were acquired consecutively and assembled subsequently.

## Results

### Formation of the central lumen of the vessel construct

Perfused vessel constructs formed in every case a stable tube and showed open and more or less rounded lumina, with complete endothelial lining in 4/6 specimens, whereas in rotated specimens the lumina were configured irregularly and partially collapsed. Endothelial lining in rotated specimens was incomplete for the most part. The intramural capillary-like network was found directly beneath the luminal and the outer (extraluminal) surface. In some regions capillary-like strains had a size and structure histologically similar to natural microvessels, in others only malformed and ectatic capillary-like lumina were found. At times the lumina were filled with apoptotic endothelial cells; this was observed in the inner region of the vessel wall, whereas close to the inner or outer surface there was less apoptosis. In rotated specimens apoptotic rate was higher than in perfused specimens (Frerich et al. submitted). Only directly beneath luminal and the outer, extraluminal surface a more dense layer of α-actin-positive cells (smooth muscle cells) was found.

### Branching from the inner lumen

**Figure 2:**
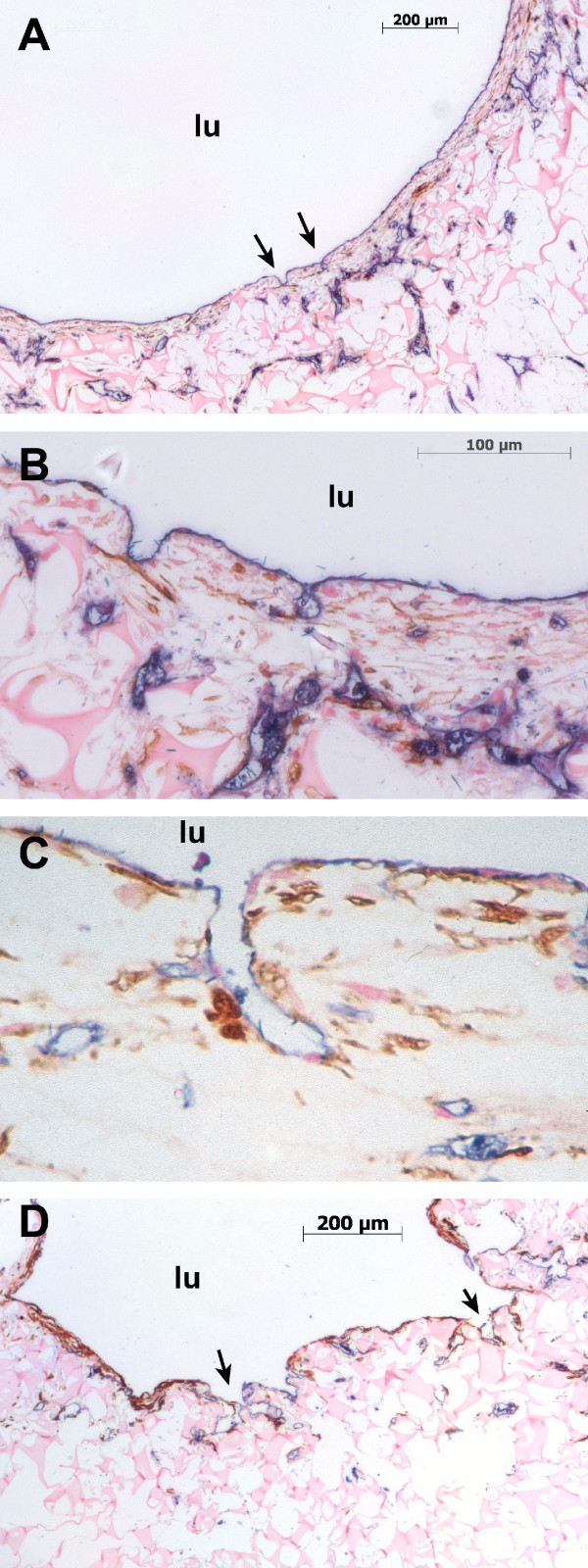
**Immunohistochemistry: Double labelling with anti-CD31 and α-actin**. Photomicrographs of specimens from perfusion culture and controls labeled with antiCD31 (Tetrazolium, blue) and anti-α-actin (DAB, brown). 2A shows a low power magnification of a cross section of an perfused specimen. Arrows demonstrate entrances from the central lumen to the capillary-like network in the subendothelial region. 2B and 2C show high power magnifications of these "entrances" or "branches". 2D represents an example of a rotated specimen, also with a luminal layer of endothelial cells, but in contrast to the perfused specimen, capillary-like network formation in the underlying region is less marked, and the "branches" represent rather empty spaces of the scaffold material lined by endothelial cells. lu = central lumen of the vessel construct.

**Figure 3:**
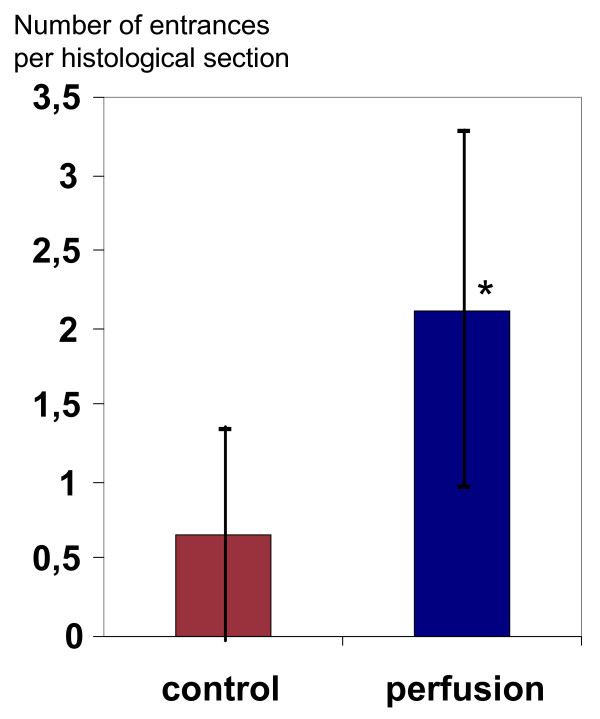
Count of entrances

The entrances branching off from the inner lumen were counted on histological cross sections (for examples see Fig. 2). Only branches lined with endothelial cells, an underlying cell layer and a tubular structure were considered as capillary branches. Two control specimens had to be dismissed, since their lumen had completely collapsed. The results are depicted in Fig. 3 and demonstrate that a more than threefold number of entrances was found in the luminal surface of perfused specimens compared with the control specimens from the rotating culture. This difference proved to be significant (p < 0,05). In perfused specimens, the branches or entrances had connected to the capillary-like network of the vessel wall. In perfused cultures, the branches had primarily a tubular vessel-like shape whereas in rotated specimens these "branches" were more often empty spaces of the scaffold lined by endothelial cells (see example Fig. 2D). There were only few capillary branches crossing the whole vessel wall.

### CLSM morphology of the capillary-like network

**Figure 4:**
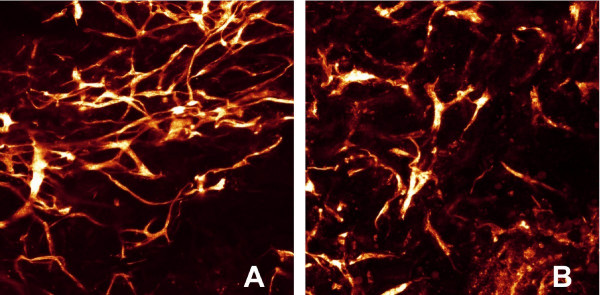
**CLSM images of perfused and rotated specimen**. Laser scanning micrographs showing the three-dimensional aspect of the capillary-like network in the luminal region of the vessel wall. In perfused specimens, the capillary-like network has a physiological, interconnected appearance (A), whereas in the rotated specimen from the same experiment (B) capillary-like structures are short and interrupted. Note the concentric organisation of capillary-like structures around the vessel lumen under pulsatile perfusion. In both cases, micrographs show the inner luminal portion. lu = central lumen of the vessel equivalent. Length of the pictures = 800 μm, maximum projection of a 60 μm deep scan, UEA-I-TRITC labelling, green excitation.

While histological sections are only two-dimensional, CLSM provides three-dimensional data and gives spatial related information about network formation. CLSM data were obtained from five experiments. In two experiments, perfused specimens showed intensive formation of an interconnected and apparently more "physiological" plexus than the counterparts from rotation culture. There was a strong orientation of the capillary-like structures around the central lumen in perfused cultures (Fig. 4A), whereas in rotated specimens capillary strains were shorter and incoherent, or formed clumsy networks (Fig. 4B). The experiment, which had been conducted with serum-reduced instead of serum free culture medium, clearly showed better results concerning network formation. In the three further pairs of experiments, the differences between perfused and rotated specimens were less marked, also due to insufficiencies of three-dimensional image acquisition. In every case, however, the perfused specimen showed more extended capillary-like network formation than the rotated one.

**Figure 5:**
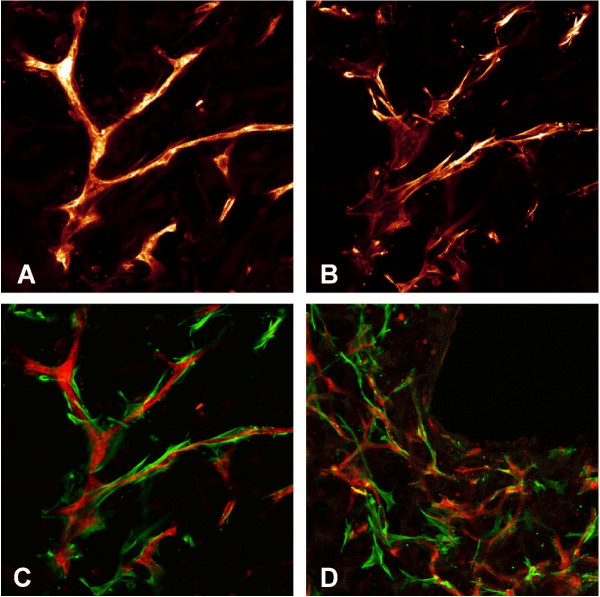
**CLSM: Double labelling with anti-CD31 and α-actin**. Demonstration of capillary-like structures labelled with UEA-I-TRITC (red) and anti-α-actin-FITC (green). A-C shows images of a single capillary-like structure, A: UEA-I-positive capillary strain, B: α-ctin-positive cells, C: combined image demonstrating the recruitment of α-actin-positive cells to capillary strains. D: low power magnification showing a part of the lumen.

Double staining with TRITC-labelled UEA1 and FITC-labelled anti-α-actin was used in order to assess vessel maturation. Immature capillaries remain in a state of plasticity as long as they are uncovered by mural cells, i.e. pericytes and smooth muscle cells. With the recruitment of mural cells (α-actin-positive cells), mediated by various cytokines, they become stable and mature (see discussion). In that way, the recruitment of α-actin-positive cells to capillary strains is a significant sign of vessel maturation in vivo and also in vitro. In perfused and rotated specimens equally the recruitment of α-actin positive cells to capillary-like structures was observed. It was striking that almost all α-actin-positive cells had contact to the capillary-like strains (Fig. 5). It might be concluded, that either smooth muscle differentiation was dependent on contact to endothelial cells or that there was a strong recruitment of all cells with smooth muscle cell differentiation to the capillary strains.

## Discussion

The problems of nutrition and vascularization have been identified as crucial parameters in tissue engineering. Recent approaches dealing with natural vessel loops included in engineered tissues were successful in enhancing vascularization as well as tissue growth in larger three-dimensional aggregates [15,16]. Consequently a further step consists in the use of vessel grafts or substitutes in order to achieve improved supply of engineered tissues. Autologous vessels continue to be considered the most favourable option, but they are by no means the ideal graft and their availability is limited. Both synthetic polymers and biogenic homologous materials have been examined in the search for suitable alternative materials. The seeding of graft polymers in vitro with human endothelial cells with a view to implanting small-diameter vascular grafts with an anti-thrombogenic autologous endothelial layer has improved patencies [17,18], but still has limitations due to the poor adherence of endothelial cells to alloplastic materials. Furthermore, these materials build a physical barrier, which prevents the long-term remodelling of the vessel substitute and leads to anastomosal intimal hyperplasia. For these reasons, the tissue engineering of vessels has been proposed as a solution [19,20]. Alloplastic vessel substitutes and tissue engineered vessel equivalents existing to date both have in common that they are designed for the purpose of a conduit and not for the development of branches. This capability is however mandatory for the application as an artificial supplying vessel for tissue engineered transplants and was therefore the aim of the present study.

In our investigations, conditions of pulsatile perfusion culture enhanced the development of vascular branches from the central lumen of the artificial vessel equivalent. Strain and fluid shear stress are known to be key factors in the regulation of vascular growth and remodelling [21]. Fluid shear stress has been shown to be an important regulator of vascular structure and function through its effect on the endothelial cell. Shear stress increases the expression of PDGF-B mRNA and of bFGF mRNA in endothelial cells [22] and also stimulates SMC function, including the release of PDGF-A [23], bFGF [24] and nitric oxide [25]. Pulsatile pressure increases the growth of SMCs [26] and also their migration and cytokine expression [27]. It can therefore be assumed that these factors are playing a role in the stabilization of capillary-like structures in vitro. The remodelling process, which is also dependent on the presence of mural cells [28], is presumably regulated by the same factors, thus providing an explanation for the improved network formation in the perfused tubes.

It has to be conceded, that there was variability in the results of the different experiments, especially in the histological appearance of the capillary-like structures and in the degree of mural cell differentiation. Furthermore, due to methodological reasons of this vessel model, it was difficult to determine exactly the pressure and the shear forces, which were applied. Therefore it is difficult to draw exact and quantifiable conclusions concerning the influence of the hydrodynamic forces. However it becomes clear, that physical parameters support tissue differentiation also on the level of the vascular system. The CLSM pictures (Fig. 4) show that perfusion culture clearly promotes the more physiological appearance of an interconnected capillary-like network, whereas the structures in a rotating culture remained short and were incoherent.

## Conclusion

The proposed concept of an artificial "feeder donor vessel" represents a possible approach to the problem of vascularization in tissue engineering and has the advantage of pointing to a solution for the problem of nutrition and oxygenation in the in vitro phase of engineering larger tissue equivalents. A further argument for this approach are the requirements in specific situations of reconstructive surgery. Following radiation the recipient bed is not able to provide sufficient vascularization, for instance. The alternative way of angiogenic growth factor administration is not an option after tumour surgery. Such in vitro models of "microvascular engineering" may also have a far-reaching impact on the conduction of pharmaceutical testing, and may in fact eventually replace animal studies.

However, basic requirements for the evolution of this concept are adequate monitoring systems. In case of "microvascular" engineering, this comprises the need for imaging and image analysis in order to gain spatial related data as well as functional data, which reveal information about the functional capacity of the engineered vessels. Even lumen containing capillary-like structures in vitro are not automatically prone to real function in terms of nutrition and oxygenation. Therefore the linkage between functional data and image analysis is needed in order to get valuable in vitro models for vascular engineering.

## Competing interests

Parts of this investigation have been submitted and published for a patent application, applicant BF. Besides this the authors declare that they have no competing interests.

## Authors' contributions

BF conceived and conducted the in vitro experiments and wrote the manuscript drafts, KZ performed the immunohistochemical staining, histomorphometric evaluations and laserscanning microscopy. AH revised and corrected the drafted manuscript. All authors read and approved the final manuscript.

## References

[B1] Auger FA, Rouabhia M, Goulet F, Berthod F, Moulin V, Germain L (1998). Tissue-engineered human skin substitutes developed from collagen-populated hydrated gels: clinical and fundamental applications. Med Biol Eng Comput.

[B2] Frerich B, Kurtz-Hoffmann J, Lindemann N, Müller S (1999). Untersuchungen zum Tissue engineering vaskularisierter knöcherner und weichgewebiger Transplantate. Mund Kiefer Gesichtschir.

[B3] Frerich B, Lindemann N, Kurtz-Hoffmann J, Oertel K (2001). In vitro vascular stroma model for the engineering of vascularized tissues. Int J Oral Maxillofac Surg.

[B4] Germain L, Remy ZM, Auger F (2000). Tissue engineering of the vascular system: from capillaries to larger blood vessels. Med Biol Eng Comput.

[B5] Kaihara S, Borenstein J, Koka R, Lalan S, Ochoa ER, Ravens M, Pien H, Cunningham B, Vacanti JP (2000). Silicon micromachining to tissue engineer branched vascular channels for liver fabrication. Tissue Eng.

[B6] Borges J, Mueller MC, Padron NT, Tegtmeier F, Lang EM, Stark GB (2003). Engineered adipose tissue supplied by functional microvessels. Tissue Eng.

[B7] Shepherd BR, Chen HY, Smith CM, Gruionu G, Williams SK, Hoying JB (2004). Rapid perfusion and network remodeling in a microvascular construct after implantation. Arterioscler Thromb Vasc Biol.

[B8] Wenger A, Stahl A, Weber H, Finkenzeller G, Augustin HG, Stark GB, Kneser U (2004). Modulation of in vitro angiogenesis in a three-dimensional spheroidal coculture model for bone tissue engineering. Tissue Eng.

[B9] Kannan RY, Salacinski HJ, Sales K, Butler P, Seifalian AM (2005). The roles of tissue engineering and vascularisation in the development of micro-vascular networks: a review. Biomaterials.

[B10] Polverini PJ (2002). Angiogenesis in health and disease: insights into basic mechanisms and therapeutic opportunities. J Dent Educ.

[B11] Brey EM, Patrick CW (2000). Tissue engineering applied to reconstructive surgery. IEEE Eng Med Biol Mag.

[B12] Nerem RM, Alexander RW, Chappell DC, Medford RM, Varner SE, Taylor WR (1998). The study of the influence of flow on vascular endothelial biology. Am J Med Sci.

[B13] Morita T, Yoshizumi M, Kurihara H, Maemura K, Nagai R, Yazaki Y (1993). Shear stress increases heparin-binding epidermal growth factor-like growth factor mRNA levels in human vascular endothelial cells. Biochem Biophys Res Commun.

[B14] Albuquerque ML, Waters CM, Savla U, Schnaper HW, Flozak AS (2000). Shear stress enhances human endothelial cell wound closure in vitro. Am J Physiol Heart Circ Physiol.

[B15] Cronin KJ, Messina A, Knight KR, Cooper-White JJ, Stevens GW, Penington AJ, Morrison WA (2004). New murine model of spontaneous autologous tissue engineering, combining an arteriovenous pedicle with matrix materials. Plast Reconstr Surg.

[B16] Hofer SO, Knight KM, Cooper-White JJ, O'Connor AJ, Perera JM, Romeo-Meeuw R, Penington AJ, Knight KR, Morrison WA, Messina A (2003). Increasing the volume of vascularized tissue formation in engineered constructs: an experimental study in rats. Plast Reconstr Surg.

[B17] Herring MB, Compton RS, LeGrand DR, Gardner AL, Madison DL, Glover JL (1987). Endothelial seeding of polytetrafluoroethylene popliteal bypasses. A preliminary report. J Vasc Surg.

[B18] Zilla P, Deutsch M, Meinhart J (1999). Endothelial cell transplantation. Semin Vasc Surg.

[B19] L'Heureux N, Paquet S, Labbe R, Germain L, Auger FA (1998). A completely biological tissue-engineered human blood vessel. FASEB J.

[B20] Hoerstrup SP, Zündl G, Sodian R, Schnell AM, Grünenfelder J, Turina MI (2001). Tissue engineering of small caliber vascular grafts. Eur J Cardio Thor Surg.

[B21] Resnick N, Yahav H, Shay-Salit A, Shushy M, Schubert S, Zilberman LC, Wofovitz E (2003). Fluid shear stress and the vascular endothelium: for better and for worse. Prog Biophys Mol Biol.

[B22] Malek AM, Gibbons GH, Dzau VJ, Izumo S (1993). Fluid shear stress differentially modulates expression of genes encoding basic fibroblast growth factor and platelet-derived growth factor B chain in vascular endothelium. J Clin Invest.

[B23] Sterpetti AV, Cucina A, D'Angelo LS, Cardillo B, Cavallaro A (1992). Response of arterial smooth muscle cells to laminar flow. J Cardiovasc Surg.

[B24] Rhoads DN, Eskin SG, McIntire LV (2000). Fluid flow releases fibroblast growth factor-2 from human aortic smooth muscle cells. Arterioscler Thromb Vasc Biol.

[B25] Papadaki M, Tilton RG, Eskin SG, McIntire LV (1998). Nitric oxide production by cultured human aortic smooth muscle cells: stimulation by fluid flow. Am J Physiol.

[B26] Watase M, Awolesi MA, Ricotta J, Sumpio BE (1997). Effect of pressure on cultured smooth muscle cells. Life Sci.

[B27] Redmond EM, Cahill PA, Hirsch M, Wang YN, Sitzmann JV, Okada SS (1999). Effect of pulse pressure on vascular smooth muscle cell migration: the role of urokinase and matrix metalloproteinase. Thromb Haemost.

[B28] Uemura A, Ogawa M, Hirashima M, Fujiwara T, Koyama S, Takagi H, Honda Y, Wiegand SJ, Yancopoulos GD, Nishikawa S (2002). Recombinant angiopoietin-1 restores higher-order architecture of growing blood vessels in mice in the absence of mural cells. J Clin Invest.

